# Powering strategies for implanted multi-function neuroprostheses for spinal cord injury

**DOI:** 10.1049/htl.2019.0113

**Published:** 2020-06-24

**Authors:** Kevin L. Kilgore, Brian Smith, Alex Campean, Ronald L. Hart, Joris M. Lambrecht, James R. Buckett, Paul Hunter Peckham

**Affiliations:** 1 Department of Orthopaedics and Department of Physical Medicine and Rehabilitation, MetroHealth System, Cleveland, OH, 44109, USA; 2 Department of Biomedical Engineering, Case Western Reserve University, Cleveland, OH, 44106, USA; 3 Research Service, VA Northeast Ohio Healthcare System, Cleveland, OH, 44106, USA

**Keywords:** biocontrol, handicapped aids, biomedical equipment, diseases, injuries, neuromuscular stimulation, neurophysiology, biomedical electrodes, bioelectric phenomena, prosthetics, biomechanics, muscle, powering strategies, implanted multifunction neuroprostheses, spinal cord injury, implantable motor neuroprosthetic systems, significant disabilities, cerebral palsy, multiple sclerosis, paralysed muscles, coordinated patterns, controlled movement, implanted system, wired multipoint implant technology, implanted multifunction neuroprosthetic systems, centralised power supply, actively cooled external recharge coil, implanted neuroprostheses

## Abstract

Implantable motor neuroprosthetic systems can restore function to individuals with significant disabilities, such as spinal cord injury, stroke, cerebral palsy, and multiple sclerosis. Neuroprostheses provide restored functionality by electrically activating paralysed muscles in coordinated patterns that replicate (enable) controlled movement that was lost through injury or disease. It is important to consider the general topology of the implanted system itself. The authors demonstrate that the wired multipoint implant technology is practical and feasible as a basis for the development of implanted multi-function neuroprosthetic systems. The advantages of a centralised power supply are significant. Heating due to recharge can be mitigated by using an actively cooled external recharge coil. Using this approach, the time required to perform a full recharge was significantly reduced. This approach has been demonstrated as a practical option for regular clinical use of implanted neuroprostheses.

## 1 Introduction

Implantable motor neuroprosthetic systems can restore function to individuals with significant disabilities, such as spinal cord injury (SCI), stroke, cerebral palsy (CP), and multiple sclerosis (MS) [1, 2]. Neuroprostheses provide restored functionality by electrically activating paralysed muscles in coordinated patterns that replicate (enable) controlled movement that was lost through injury or disease.

The common components of motor neuroprostheses (stimulating electrodes and sensors) place unique requirements on system design when contrasted with other active implantable medical devices such as pacemakers and spinal cord stimulators (SCSs) [3]. For example, the physical location of the stimulating electrodes in a motor neuroprosthesis is typically anatomically distributed, usually placed in multiple locations throughout one or more extremities, whereas the region of activation for other devices is localised (e.g. heart for pacemakers, spinal cord for SCS, brain for deep brain stimulators etc.). Furthermore, in general, it is not possible for a single multi-contact stimulating electrode to successfully activate all of the target muscles/organs in a motor neuroprosthesis. In addition, sensors used in a motor neuroprosthesis tend to be placed in the periphery as well, often remote to the location of electrical stimulation [3]. Given the unique requirements of motor neuroprostheses, it is important to consider the general topology of the implanted system itself [4]. The commonly adopted topologies utilised for other active implantable medical devices are not necessarily optimum for motor neuroprostheses [3, 5].

**Fig. 1:**
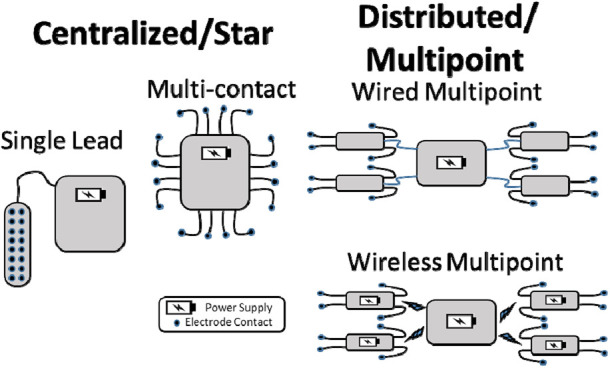
Comparison of topologies that can be used for implantable systems. For comparison, each configuration shown delivers 16 channels of stimulation. The single lead topology, such as utilised for SCS, is the most common but cannot be used for motor neuroprostheses where electrodes must be distributed

In general, the topology of a neuroprosthesis can be either ‘distributed’ (or ‘multipoint’) or ‘centralised’, as shown in Fig. 1 [4]. More specifically, for a fully-implanted system that includes electrodes, stimulators, sensors, processing capacity, and powering, all of these features can be considered separately with respect to a distributed or centralised architecture [3]. We examine the advantages and disadvantages of this approach in the following paragraphs.

The wired multipoint topology has distributed stimulator and sensor circuitry with distributed processing, but has centralised power. It consists of a network of essentially equal nodes (or modules), with the exception that one node contains all of the powering for the entire network. The key advantage of this topology, when compared to the wired star topology (‘centralised’ – base topology for most implanted neuromodulation systems where all leads are connected to a single centralised device), is the significant reduction in lead routing and lead length required. Both the wired and wireless multipoint topologies address the problem of ‘maxing out’ a central processor in terms of capacity for physical connections, capacity for computational processing, and capability to incorporate new features into the system. The latter is a key advantage for the multipoint systems. The total capacity of the network can be expanded by adding additional modules without disrupting the configuration of the existing modules. New functions can also be added, such as adding a new modality of sensor information, by incorporating these functions in a new module that is capable of communicating on the network.

The advantages of the wired multipoint topology over the wireless multipoint topology are based on the power distribution network. First, the wired multipoint topology has a central power supply. As a result, the user only needs to place a single external charging coil at a single well-defined location. This allows the ‘power module (PM)’ to be designed for optimum efficiency regarding the transcutaneous coupling for charging. In the case of the wireless multipoint topology, it may be necessary for the user to place multiple external charging coils over the various modules, depending on their location throughout the body. Finally, given that the power storage will degrade and eventually need to be replaced, the wired multipoint topology minimises the difficulty in performing this replacement, since only a single module (and potentially a single connection) needs to be replaced.

The obvious disadvantage of the wired multipoint topology is that it requires physical connections between each module to transmit power (and to a lesser extent, data). This increases the complexity of the surgical installation since leads must be tunnelled between each module. Connector design becomes critical, although nearly every topology requires connections due to the need to place electrodes in areas too small for modules (e.g. the palm of the hand).

Based on these considerations, we selected the wired multipoint topology as the fundamental basis for the design of a new motor neuroprosthetic system, referred to as the networked neuroprosthesis (NNP) system. In this Letter, we focus on the critical aspect of the design, which is the central power supply. The wired multipoint topology places significant requirements on this component.

## 2 Design

We developed functional and technical specifications for the NNP system based on our analysis of the anticipated clinical applications and our clinical experience in the deployment of multiple neuroprosthetic systems providing motor control [6–11]. The NNP system is a fully modular wired multipoint distributed concept that meets the desired design inputs. The specifications for the NNP system were based on the features necessary to restore function to individuals with paralysis, including not only SCI, but other diseases such as stroke, MS, and CP [2]. However, the general concept of a wired multipoint system extends to many clinical applications beyond motor neuroprostheses, including systems of sensors where sensors must be placed in remote locations in the body [3].

**Fig. 2:**
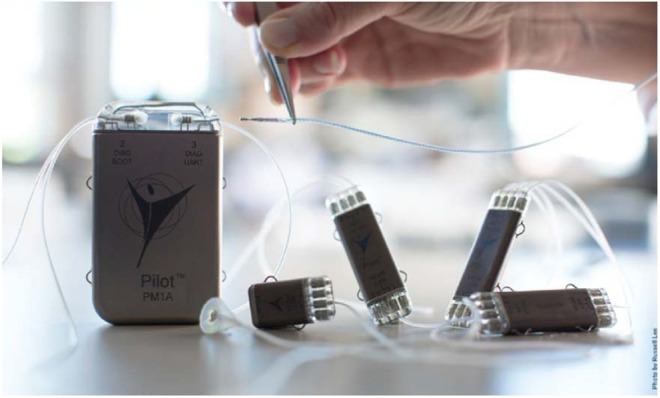
Implantable components of the NNP system. The package on the left is the PM containing three Li-ion rechargeable cells. Smaller packages are remote modules. A network cable connects all components for communication and powering

Our first implementation of the NNP system concept, described in this Letter, included the capacity for electrical stimulation via muscle- and nerve-based electrodes, myoelectric signal sensing, three-axis acceleration, and temperature sensing. The specific implanted components of the first incarnation of the NNP are shown in Fig. 2, and include a PM, a four-channel pulse generator module (PG4), a two-channel biopotential recording module (BP2), network cabling, and stimulating and recording electrodes. The modules can be connected together as needed for each clinical application, including multiple PG4 and BP2 modules, allowing the system capabilities to be tailored to the desired clinical implementation. Each module contains local processing capabilities to minimise the communication between modules and can be programmed through a transcutaneous wireless link in the PM. The modules are connected to the network using a single two-conductor lead that distributes power and provides a data communication link between each module. Network communication utilises the industrial standard ‘controller area network’ protocol over a proprietary direct current (DC)-balanced hardware layer. The NNP derives its power from lithium (Li)-ion batteries in the PM that are rechargeable through a single transcutaneous inductive link.

The NNP system is fully implanted but requires an external charger to inductively recharge the Li-ion batteries in the PM. A single external component, the control tower (CT) serves as the clinical programming link, the user's system status interface, and as the user's charger. The CT can be connected to a computer via universal serial bus for programming the entire implanted system.

A common package design is utilised for both stimulating and sensing modules, collectively referred to as ‘remote modules’. This package design allows multiple network connections to be made into or out of a single module, greatly simplifying installation in the body and maximising architectural flexibility. Remote modules are small enough to be placed in most areas of the body, including the forearms. The PM utilises similar packaging materials, but it is larger to accommodate significant power capacity through Li-ion rechargeable batteries. System programming is performed through a bi-directional wireless link contained in the PM and CT.

**Fig. 3:**
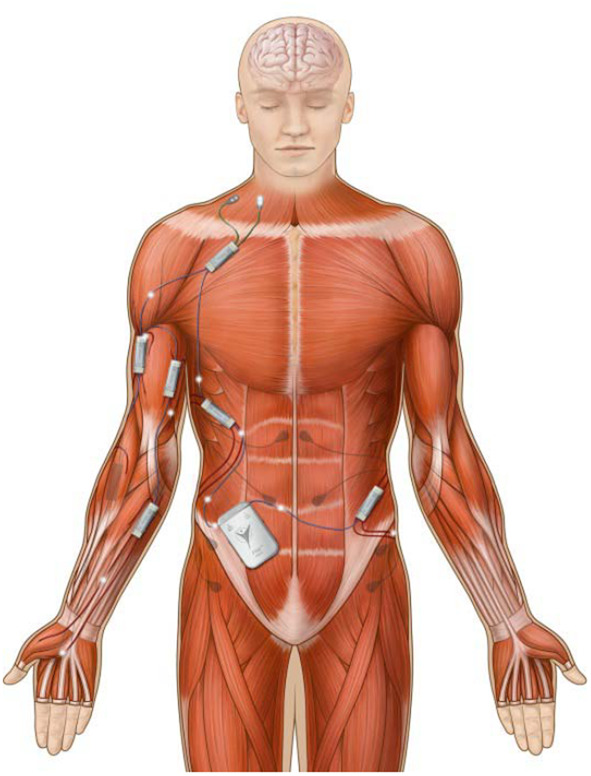
Example of NNP as arranged in the body to provide hand grasp, overhead reach, and trunk posture control for an individual with cervical SCI. Implanted components are shown in their approximate location. An external charging coil is placed on the skin over the PM (abdomen) to recharge the batteries

A typical distribution of modules in the body is shown in Fig. 3. This example, in which the NNP system is configured to provide upper extremity and trunk function for SCI [11] utilises one PM, five PG4 modules (20 stimulating electrodes), and two BP2 modules (four recording electrodes). The placement of modules and electrodes is based on our past experience with implanted neuroprosthetic systems for SCI.

### 2.1 Power module (PM)

The PM is an implanted module that has two key functions. First, the PM houses a rechargeable Li-ion power supply for the entire NNP implanted system, along with the required recharging link and circuitry. Second, the PM contains a wireless link using the MedRadio band for transcutaneous communication and system programming. The PM connects to the network of modules via a wired connection (network cable). The PM places charge-balanced alternating current (AC) power onto the network cable for distribution to all remote modules. During functional operation, the PM primarily functions as the power source for the entire implanted NNP system. However, due to the significant processing power contained in the PM, it is capable of performing signal processing and data storage in support of the remote module functions when required. The PM is always active (except under the emergency shut-down condition), but can be placed into and taken out of a low-power ‘sleep’ mode by the user. Key features of the PM are summarised in Table 1.

**Table 1:** Key features of NNP PM

Functional specifications
lifetime at least two years before battery recharge capacity or shelf-life is exhausted
full recharge in <16 h
enclosure sized to be subcutaneously implanted in the abdomen or chest in a full-size adult
biocompatible enclosure
single cable network connection to remote modules
replacement achieved via a single disconnection and single incision

**Table:** 

Technical specifications
provides a bidirectional transcutaneous data-link to the external CT – MedRadio compliant wireless
ARM7 core, 32-bit microcontroller (can be used in 16-bit mode for greater code density) with ‘on-the-fly’ adjustable clock frequency (computation versus power consumption)
expansive non-volatile memory
RTOS – micrium
4 MB flash memory for data logging
real-time clock (date and time)
inductively-coupled recharging and real-time powering link
magnetic (safety) failsafe reset
on board temperature sensing (multiple sensors) and three-axis accelerometer
Li-ion rechargeable cells (3), with safety supervision and fuel gauging (600 mAh capacity)
recharge limited to safe transcutaneous and implant heating standards
inductive coupling distance of ∼3 cm
hermetic enclosure

The PM circuit components are placed on a rigid-flex (i.e. FR4-polyimide) printed circuit board (PCB) that folds inside a polymer nest. The top portion of the PCB connects to the feedthroughs. The bottom portion of the PCB connects to the internal coil. The PM utilises a 32-bit ARM-7 microprocessor (NXP Semiconductor LPC2129). The processor runs a real-time operating system (RTOS; Micrium µC/OS-III). The PM circuit also includes a wireless transcutaneous communication circuitry based on the 402–405 MHz Medical Implant Communication System (MICS) band at 100 kbit/s (TI CC1101 radio transceiver).

Network power is generated by the three rechargeable batteries located in the PM. The PM provides DC-to-AC power conversion. All power transferred between modules is via AC, which greatly reduces any DC leakage in the case of a network cable breakage. Battery charging occurs independently of the power network operation.

The PM has a grade 23 titanium (Ti) case with a Ti lid through containing the feedthroughs for making the network connections and wireless antenna. The case is 68 mm × 47 mm × 14 mm and has no sharp edges or other features that would encourage tissue ingrowth (and thus make removal and/or replacement more problematic). A polymer header (Tecothane backfilled with silicone rubber) holds the connections and antenna.

Three identical Li-ion rechargeable cells are connected in parallel to provide the necessary power for the NNP system. Each cell is an implantable grade (Quallion LLC QL0200I-A) prismatic Li-ion cell, 200 mAh, 3.6 V nominal voltage. Each cell is 5.5 mm × 17 mm × 35 mm and weighs 8 g. These cells have excellent characteristics for implantable medical devices, including the capacity to be completely discharged to zero volts and then recharged without significant loss of capacity [12–14].

Although charging is microprocessor-controlled, the charging circuitry limits the peak charging current to 100 mA. Furthermore, the circuitry clamps the charging voltage to a maximum of 4.1 V to stay within the battery's safe operating envelope. Charging is shut down by default on power-up and must be actively engaged by the microprocessor to operate. Charge current can be shut down or adjusted at any time by the microprocessor.

Each battery is continuously monitored by a battery fuel gauge (Texas Instruments BQ27200 Li-ion). The battery fuel gauge reports 22 parameters that reflect the past and present battery operating status to the microprocessor. Raw values such as voltage and current are provided in addition to refined parameters such as ‘at-rate’ time remaining to discharge, per cent capacity remaining, and last measured discharge.

A polymer nest inside the PM case holds the batteries and circuitry in place and provides the structure for placement of the copper recharge coil. This ensures that the flex-circuit areas of the PCB do not support any mechanical load except for the small weight of the PCB itself. The nest also provides a secure mounting location for a temperature sensing thermistor to ensure that it remains in intimate contact with the enclosure body.

The PM contains a magnetically activated reed switch for the emergency shut-down of the entire NNP system. In a strong magnetic field (i.e. magnet present), this switch initiates a failsafe shutdown function that will de-energise all of the PM circuitry.

The PM contains a three-axis accelerometer and has a thermistor for enclosure temperature sensing. The thermistor can be used in a charging feedback loop to minimise recharge time while maintaining the enclosure temperature at a safe level.

The PM is designed to be implanted in the torso, typically either chest or abdomen. This anatomical location has been used in our previous generations of neurostimulators used in the lower extremity [15], and these portions of the body can accommodate the PM package size. This location also allows convenient access for recharging through an inductive link, and easy surgical exposure for replacement. The PM is designed for easy surgical replacement, and replacement of the PM is an anticipated and expected event based on the eventual depletion of the Li-ion batteries storage capacity. PM replacement is accomplished through a single small incision, disconnection of the network segment connection, and replacement with a new PM.

### 2.2 External CT/charger and recharge coil

An external CT unit provides the recharging capability for the NNP. The CT has an inductive link for charging via an external coil and also has a bidirectional wireless communication link between the implanted NNP components and external systems (via MedRadio) for system programming. The battery charging/powering portion of the CT uses a loosely coupled inductive power transfer link. Inductive coupling is optimised for power transfer efficiency through the skin and into the PM and is designed to maintain safe transcutaneous power transfer based on the recharge rate established through recharge testing. The CT provides the status of battery charge and related information for the user. However, it should be noted that the CT is not required for regular functional use.

The external recharge coil safely provides the appropriate time-varying magnetic field required to recharge the PM. The recharge coil is externally applied over the site of the implanted PM. A thermistor is used to measure the temperature of the coil/skin interface. The recharge coil has an asymmetric shape so that it can only be applied with the appropriate surface against the skin. The 3.5 kHz drive voltage to the recharge coil is set in hardware such that the coil temperature at the coil/skin interface does not exceed 40°C.

## 3 Test methods

One potential disadvantage of the wired multipoint topology with a centralised power source is that the single large battery requires relatively high current for recharge, introducing a risk of overheating that must be mitigated. To mitigate this risk, we performed a series of recharge tests to set a maximum limit for charging output that maintained the components below the established standards for safe tissue heating (see British Standard EN 45502-2-2:2008). The charging limit is implemented in the electronic hardware of the CT charging circuit and the temperature limit is implemented in software. As a result, the safe recharge is ensured through software with a hardware failsafe backup.

Heating of the PM has several potential causes:

- Eddy currents when the external coil is on and near the PM or other active component.
- Current regulators in the charging circuitry.
- Implant circuitry (e.g. microcontroller, radio) when the PM is active.
- Network drive when the remote modules are active.

If the magnetic field is larger than necessary, the eddy-current heating will predominate, and the temperature rise will increase even as the batteries enter constant voltage charging and the devices utilise less of the magnetic field. To minimise temperature rise, the magnetic field sensed by the PM coil should be the minimum necessary to support the desired charging current. This can be achieved by increasing the space between the charge coil and the implant, or by reducing the drive voltage of the charge coil.

### 3.1 Recharge heating characterisation in air

To characterise recharge heating, we conducted a test in the air. The recharge heating test was performed using an experimental set up that includes the PM enclosure and receive coil, the external charging coil, drive circuitry, and thermocouples. The PM enclosure was placed flat on a non-conductive surface with a thermocouple attached to the top surface to record case surface temperature. The internal coil of the PM was monitored via leads exiting the case. This provided a direct measure of the received power and current developed in the internal coil, enabling direct calculation of the expected rate of recharge for each test condition. The inductive recharge coil was placed 3 cm above the top surface of the PM case and was suspended in place with air between the bottom surface of the coil and the top surface of the PM enclosure. The test was performed at room temperature with no forced air circulation around the components. We hypothesised that this configuration represented a worst-case in regard to the heating of the case because the perfusion of living tissue provides considerable heat transfer in contrast to non-circulating air [16, 17].

We determined the power transfer across the inductive link formed by the transmit coil and PM, which contains the receive coil. The inductive link operates as a series of tuned primary and a series of tuned secondary. The resonant frequency of the link was ∼3.5 kHz. The transmit coil/capacitor was driven by the coil drive amplifier (square wave) at the resonant frequency. The coil drive amplifier DC voltage and current were recorded during the experiments. The AC in the transmit coil was measured with a current probe (Tektronix AM503B) and recorded. The coil current was set by the coil drive amplifier DC voltage. The output of the receive coil was rectified and filtered. This DC output was connected to a variable resistive load and measured on an oscilloscope.

The coil drive voltage was set to develop a range of received power levels from 200 to 2000 mW. Starting at the highest charge rate, the power to the inductive coil was delivered continuously while the temperature was monitored and recorded. When the temperature of the PM case increased by 4°C above ambient, the test was terminated and the results recorded. The components were allowed to cool to room temperature and the test was repeated with a lower recharge current.

## 4 Results

### 4.1 Recharge heating characterisation in air

**Fig. 4:**
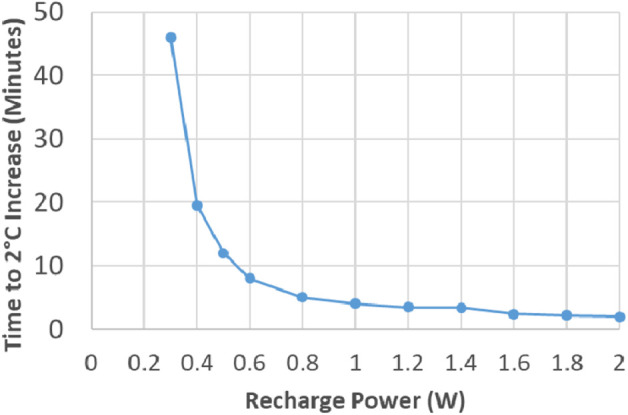
PM case heating as a function of time for different levels of received power, from 200 mW (lowest line) up to 2 W (left-most line). At 200 mW, the temperature threshold is never reached (test extended for 24 h to verify)

The results of the recharge heating test are shown in Fig. 4. It was determined that a received power level of 200 mW enabled the coil to continuously recharge without excessive heating of the PM case or the external coil. At a received power of 200 mW, the PM case temperature increased 1.2°C over the ambient temperature in the first 30 min of recharging and then plateaued. At 300 mW received power, the PM case exceeded the 2°C increase after ∼1 h of recharging.

Based on these results, the CT was designed to supply a maximum received power of 200 mW at the PM. This is accomplished by setting the maximum coil drive amplifier output to 4.7 V_DC_ and requiring the separation between the external coil and PM to be 3 cm or more. This minimum distance is established by adding padded spacers to the external coil to maintain the appropriate minimum distance. Assuming a 70% depth of discharge, a full recharge could be realised in ∼14 h.

### 4.2 Limitations of the current realisation of the NNP

As part of the early feasibility investigational device exemption (NCT #0232965), we had identified the recharging process as a key area for assessment with each subject, with the goal of identifying the most desirable and practical methods of recharge. During initial testing with the first subject, we found that the recharge rate had to be limited to a ∼12 mA recharge rate due to PM heating, requiring ∼14 h to fully recharge. Given a battery voltage of 3.7 V, this only represents 44 mW going into the batteries. We estimated that the received power by the PM was ∼340 mW at these conditions, which is consistent with the hypothesis that the maximum received power measured in air (200 mW) was a conservative estimate. After these tests, we reduced the idle power consumption of the PM from 50 mA down to 15 mA by using a wake on radio functionality and idle the microprocessor whenever possible. This allowed a much larger percentage of the received power to be used to recharge the batteries.

**Fig. 5:**
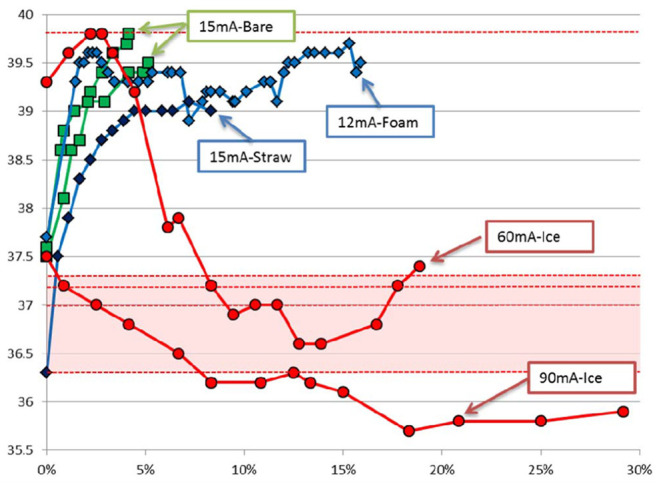
Comparison of percentage of full recharge versus PM temperature during recharge at different rates and different conditions. The recharge rate in mA is indicated for each condition. Bare = coil placed directly on the skin. Foam = insulating foam placed between coil and skin. Straw = placemat of open straws placed between coil and skin (provides passive air flow). Ice = thin ice pack placed between coil and skin. In the 90 mA recharge rate, the ice was placed on the skin to pre-cool the tissue before initiating recharge. We were unable to make repeated measurements due to the extended length of time required for each test and for the skin and device to return to a normal resting temperature

Our first subject desired a much more rapid recharge time and was not concerned about the size of the external coil and enclosure. We tested a variety of cooling techniques and recharge rates, as shown in Fig. 5. We determined that the only way to substantially reduce the recharge time was with active cooling around the external coil. We found that by placing an ice pack on the skin over the PM, we could deliver 90 mA recharge current or a 6-fold increase over the maximum recharge current without active cooling. If we placed the ice pack over the region for a few minutes prior to starting the charging process, we could cool the tissue to the depth of the PM and can cool the PM itself during recharge (see 90 mA-Ice curve in Fig. 5).

**Fig. 6:**
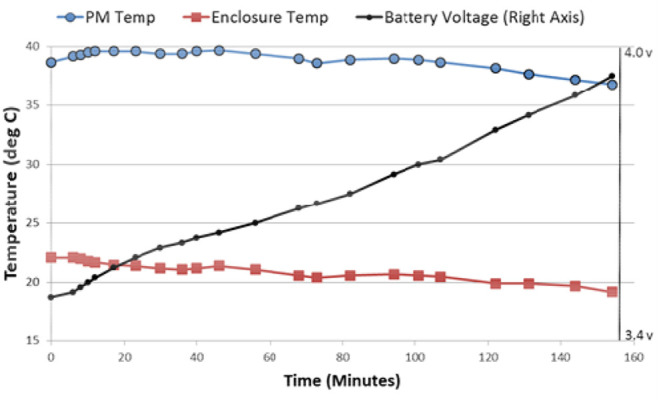
Results of the water-cooled recharge coil enclosure for the NNP system. The maximum PM temperature was 39.7°C, corresponding to a PM/tissue temperature of 38.7°C. The water-cooled enclosure has the effect of cooling the tissue down to the depth of the PM, as indicated by the steady decrease in PM temperature after ∼40 min of recharge. This data was obtained with a 50 mA recharge rate per battery, which corresponds to a full recharge in a 3.5 h period

Ice packs are very inconvenient and not safe for long term use. Therefore, we developed an external enclosure that fits around the coil, provides active cooling, and protects against any direct contact with the external recharge coil. The coil is cooled using a Peltier-based cooling pump (ThermaZone™). Each pump has a maximum cooling of 4°C and used distilled water to pump through tubing surrounding the recharge coil. In actual use, the coil was never cooled below 15°C even at the maximum setting. There was no risk of skin damage related to the cooling. As shown in Fig. 6, the actively-cooled recharge coil made it possible to fully recharge the implanted NNP system in <3 h. This allowed the subject to recharge in the morning or evenings when he has an aide available to help with the positioning of the external coil.

## 5 Discussion

Implanted neuroprosthetic systems have the potential to provide significant functional enhancement for individuals with paralysis and other neurological disorders. However, proper system design and topology are required to meet the unique needs of these systems, particularly the fact that stimulating electrodes and sensors need to be placed throughout the body. We present the rationale for the use of a wired multipoint topology as the fundamental basis for the design of such systems. We developed an NNP system based on this concept. A key feature of this system is that it utilises a centralised power source. This allows for a single site for a recharge which is required for daily practical use. Furthermore, when the power supply reaches the end of life, surgical replacement requires only the replacement of the single power supply component. However, as we demonstrate here, the single site of recharge presents the risk of overheating if the rapid recharge is to be achieved. Our work demonstrates that rapid recharge can be achieved through active cooling of the recharge coil and tissue surrounding the implanted power supply.

The PM of the NNP contains a thermistor placed in direct contact with the titanium case closest to the surrounding tissue, which serves to track the temperature of the PM during recharge and active use. This thermistor effectively measures the inner capsule temperature, which is a combination of the ambient electronics temperature and the inner surface of the titanium capsule. This feature proved to be invaluable in our initial human testing; demonstrating that cooling the skin on the surface could effectively cool the tissue down to the depth of the implanted PM (∼2–3 cm). This allowed us to increase the recharge rate by a factor of six. In the future, it may be possible to utilise the PM temperature as the primary control signal for a temperature-based closed-loop recharging algorithm.

It should be noted that the received power of 200 mW, which was determined through experiments in air, was considered to represent a conservative requirement. First, the perfusion in the body provides a significant buffer for temperature rise within the body. In practice, we could maintain a received power of in excess of ∼300 mW without overheating in our first subject. Second, there is considerable evidence in the medical literature that the body can tolerate temperatures as high as 45°C (8°C over normal surrounding temperature) for at least 30 min [17–19]. Given these features, it may be possible to further increase the recharge rate for these systems.

In the clinical use of the NNP, we have found that the cooled coil recharge system feasible for practical daily function. Specifically, users of the neuroprosthesis typically recharge in the morning or evenings while sitting in their wheelchair. Since the recharge time takes <3 h, they establish a recharge ‘station’ for regular home use. Future development can create a more efficient charging station that is more easily portable for travel. However, our work demonstrates that active cooling is a practical approach to reduce heating of implanted neuroprosthetic components during recharge. The results we present here are specific to our implanted NNP system, which provides a motor function for people with SCI. There are many other factors that may affect the applicability of our results and our approach to other implanted devices.

## 6 Conclusion

The wired multipoint implant technology is practical and feasible as a basis for the development of implanted multi-function neuroprosthetic systems. The advantages of a centralised power supply are significant. Heating due to the recharge can be mitigated by using an actively cooled coil. Combining this approach with improved efficiency in PM idle power, the time required to perform a full recharge was reduced from ∼14 h to ∼2.5 h. The cooling approach has been demonstrated as a practical option for regular clinical use of implanted neuroprostheses.
